# Highly Specific Chemiluminescence Immunoassay for the Determination of Chloramphenicol in Cosmetics

**DOI:** 10.1155/2019/7131907

**Published:** 2019-06-20

**Authors:** Qiyan Li, Riran Zhu, Jun Li, Xiaobing Wang, Lihua Xu, Yanshen Li, Peng Li

**Affiliations:** ^1^Health Food and Cosmetics Laboratory, Shandong Institute for Food and Drug Control, Jinan 250101, China; ^2^Faculty of Pharmaceutical Sciences, Affiliated Hospital of Shandong University of Traditional Chinese Medicine, Jinan 250012, China; ^3^College of Life Science, Yantai University, Yantai 264005, China; ^4^Key Laboratory of Quality & Safety Control for Milk and Dairy Products of Ministry of Agriculture and Rural Affairs, Institute of Animal Sciences, Chinese Academy of Agricultural Sciences, Beijing 100193, China

## Abstract

A direct and highly specific chemiluminescent enzyme-linked immunosorbent assay (CL-ELISA) method for monitoring chloramphenicol (CAP) in cosmetics has been developed. The anti-chloramphenicol antibody (mAb) adopted in this work for direct immunoassay could bind to CAP specifically, with negligible cross-reactivity (CR) (less than 0.01%) with most CAP analogues, including structurally related thiamphenicol (TAP) and florfenicol (FF). The limit of detection (LOD), measured by IC_10_, was 0.0021 ng mL^−1^. The detection range (IC_20_-IC_80_) was ranged from 0.00979 to 0.12026 ng mL^−1^. In spiked cosmetics samples, mean recoveries ranged from 82.7% to 99.6%, with intraday and interday variation less than 9.8 and 8.2%, respectively. Moreover, with the help of HRP-labeled anti-CAP mAb, the method could be processed in fast direct immunoreaction mode. This CL-ELISA method could be applied for specific, rapid, semiquantitative, and quantitative detection of CAP in cosmetics, facilitating the precise quality control of CAP contamination.

## 1. Introduction

**Figure 1:**
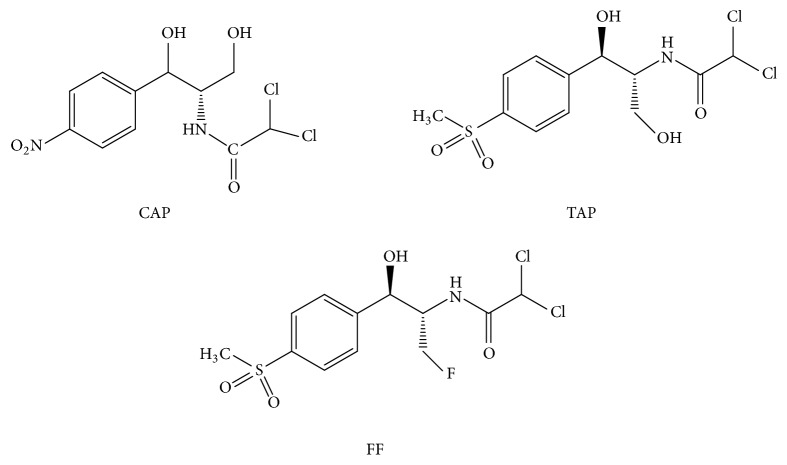
Chemical structures of CAP, TAP, and FF.

Chloramphenicol (CAP) is a member of amphenicol family of antibacterial agents, and they were produced by* Venezuelan streptococcus *(structure shown in Figure 1) [1, 2]. Chloramphenicol is usually performed as a broad-spectrum antibiotic with effective antimicrobial activity. It can be usually against a spread variety of Gram-positive and Gram-negative bacteria, including the anaerobic organisms [3]. Due to the high activity, chloramphenicol is widely applied in livestock, aquiculture, and cosmetics industries for the prevention and treatment of bacterial infection. In recent publications, chloramphenicol was reported to be used for the treatment of epifolliculitis, acne, and other skin condition. The chloramphenicol was also widely adopted in cosmetics as an antiseptic to inhibit the growth of microorganisms [4, 5].

However, recent investigations showed that amphenicol antibiotics exhibited potential adverse effects to human health [6, 7]. Therefore, the applications of chloramphenicol are banned in many countries, including EU members, USA, and China [8–11]. Although there are official regulations for these antibiotics, chloramphenicol is still illegally added to cosmetics, due to its low cost and excellent antibacterial effect. In order to ensure the safety of cosmetics and keep the human health, it is emergent to develop sensitive, reliable, and available methods for chloramphenicol detection at tracing levels in cosmetics samples.

There were numerous analytical methods reported for CAP and related antibiotic detection, such as high-performance liquid chromatography (HPLC) [12], gas chromatography (GC) [4], and liquid chromatography-tandem mass spectrometry (LC-MS/MS) [13, 14]. However, these methods mainly require expensive instruments, complicated pretreatment procedures, and well-trained staffs, and they were not suitable for fast screening large number of samples. Immunoassays mainly based on antibody-antigen specific recognition were adopted for high-throughput and rapid screening of target analytes. Chemiluminescent substrate with high-sensitivity character could be employed in a direct chemiluminescent enzyme-linked immunosorbent assay (CL-ELISA) platform. Compared to conventional colorimetric protocols, the sensitivity of immunoassays could be improved by at least 2~3 times with chemiluminescent as the substrate.

**Figure 2:**
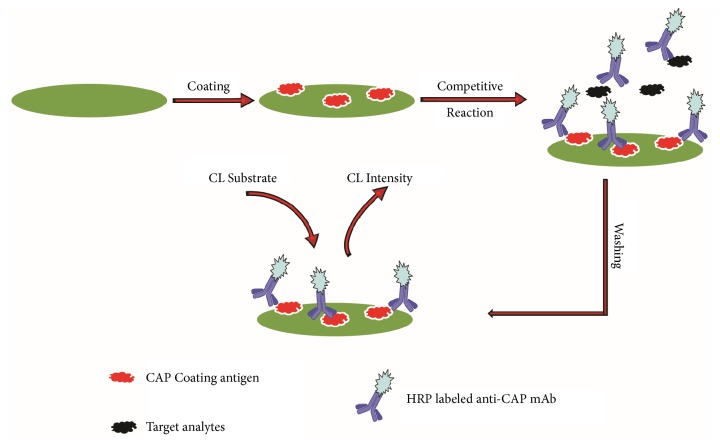
Schematic of the CL-ELISA method.

Previous literatures about immunoassay detection of CAP cannot distinguish CAP from its analogues of the amphenicol family (Figure 1), including thiamphenicol (TAP) and florfenicol (FF), because of the high-broad specific antibodies [15–17]. In these researches, it is difficult to determine whether the contamination CAP, its analogues, or sum of them when a positive result. In this research, we employed a high-specific anti-CAP monoclonal antibody (mAb) which can bind to CAP specificity and negligible CR values for most analogues tested. On the basis of this very mAb, a highly specific and direct CL-ELISA method was developed for the determination of trace amounts of CAP in cosmetics. This developed protocol does not need complex indirect conjugation reaction with HRP-labeled secondary antibody. With chemiluminescent substrate, the sensitivity could be improved significantly (schematic diagram shown in Figure 2). With the use of HRP-labeled anti-CAP mAb, the assay was conducted in fast direct immunoreaction mode without complex immunoreaction procedures. This CL-ELISA method can be applied for specific, rapid, semiquantitative, and quantitative detection of CAP in cosmetics, and this work will contribute to the quality control and regulation of CAP.

## 2. Materials and Methods

### 2.1. Chemicals and Reagents

Chloramphenicol (CAP), ciprofloxacin (CIP), florfenicol, (FF), penicillin (PEN), ractopamine (RAC), salbutamol (SAL), sulfadiazine (SUL), and thiamphenicol (TAP) standards were purchased from Sigma Aldrich (99% purity, St. Louis, MO, USA). CAP-conjugated antigen and HRP-labeled CAP antibody were obtained from ZeYang Co. (Beijing, China). SuperSignal chemiluminescence substrate solution was purchased from Thermo Fisher (USA). Milli-Q Synthesis system was obtained from Millipore (Bedford, MA, USA) for water purification. Other reagents (analytical grade) were purchased from Beijing Reagent Corp. (Beijing, China).

### 2.2. Equipment and Instrumentation

Costar white opaque 96-well polystyrene microtiter plates (Costar Inc., Milpitas, CA, USA) were used in this work. SpectraMax M5 microplate reader was obtained from Molecular Devices (CA, USA). MIKRO 22R centrifuge was obtained from Hettich Laborapparate (Tuttlingen, Germany).

### 2.3. Buffers and Solutions

For this developed CL-ELISA assay, different buffers and solutions were prepared for immunoassay.

Phosphate-buffer saline (PBS, 0.01M, pH 7.4): 8.0 g of NaCl, 2.9 g of Na_2_HPO_4_, 0.2 g of KH_2_PO_4_, and 0.2 g of KCl were dissolved in 1 L of deionized water.

Blocking buffer (pH 7.4): 0.5% casein in 0.01 M PBS.

Coating buffer (pH 9.6): 1.59 g of Na_2_CO_3_ and 2.93 g of NaHCO_3_ were dissolved in 1 L of deionized water.

Enzyme dilution buffer (pH 7.4): 5% fetal bovine serum in 0.01 M PBS.

Washing buffer (PBST): 0.01% Tween-20 in 0.01 M PBS.

### 2.4. Competitive Chemiluminescent ELISA (CL-ELISA) Procedure

Costar white opaque 96-well polystyrene microtiter plates were adopted for immunoassay reaction. 100 *μ*L of CAP-conjugated antigen was dissolved in coating buffer at a final concentration at 0.00042 ng mL^−1^ which was added and incubated at 4°C for 20 h for coating. After washing with washing buffer for three times, blocking buffer (150 *μ*L per well) was added to each well and incubated to occupy excess active sites at 37°C for 1.5 h. The coated plates were stored at 4°C before use.

For CAP analysis, the coated microtiter plates were preconditioned at room temperature for 30 min. Then, 30 *μ*L of processed samples or CAP standard and 70 *μ*L of HRP-labeled anti-CAP antibody (2.46 ng mL^−1^) diluted by enzyme dilution buffer were added to each well in turn. The plates were incubated at 37°C for 30 min for direct competitive reaction. After washing the plate with washing buffer for five times, SuperSignal chemiluminescence substrate solution (100 *μ*L/well) was added and the chemiluminescence intensity of each well was measured with a SpectraMax M5 microplate reader.

### 2.5. Standard Curve

Ten different concentration levels were tested for the evaluation of calibration curves. The highest concentration was at 1000 ng mL^−1^, and the following concentrations were serially diluted by one-third of the previous one. Each concentration was run in three times according to the CL-ELISA protocol.

B/B_0_ is defined for the chemiluminescence ratio of the results. Here, B stands for the chemiluminescence intensity of different CAP standard concentration, and B_0_ stands for chemiluminescence intensity without CAP standard in each test.

Standard curves were evaluated by plotting B/B_0_ at different CAP standard concentration and fitting the data to the following four-parameter logistic equation using Origin (version 7.5, OriginLab, Northampton, MA, USA):

(1) $$\begin{array}{c} Y=\left\{\;\frac{\left(A-D\right)}{{\left[1+\left(X/C\right)\right]}^{B}}\;\right\}+D \end{array}$$

In this equation, A stands for the upper asymptote (chemiluminescence ratio B/B_0_ without CAP standard). B is the slope of the curve at the inflection point. C, representing 50% of the maximum absorbance, is the X value at the inflection point. And D is the lower asymptote (background signal).

### 2.6. Sample Preparation for CL-ELISA Analysis

Cosmetics samples (2.00 g ± 0.02 g) were weighed precisely and transferred into 50 mL polypropylene centrifuge tubes. Then, 20 mL of PBS was added and vortexed for 30 s, and then ultra-sonication for 3 min for the extraction of CAP. Each sample was centrifuged at 12000 g for 10 min at 4°C, and the supernatant was collected and filtered through a 0.22 *μ*m membrane. Thirty microliter aliquots of the supernatant of each sample were added to a coated 96-well plate for analysis according to the developed CL-ELISA procedure.

### 2.7. Accuracy and Precision

Negative primer lotion cosmetics samples were previously confirmed by UPLC-MS/MS analyses. Then each sample was spiked with CAP standard at three different concentrations of 5, 20, and 100 ng L^−1^. Analysis was processed according to the developed CL-ELISA protocol with five replicates each (n = 5). The concentrations of samples were calculated according to the calibration curve, and the accuracy and precision were evaluated on the basis of recoveries of all the samples.

## 3. Results and Discussion

### 3.1. Assay Specificity

The specificity of the method was assessed by evaluating the extent of cross-reactivity (CR) with structurally related compounds of CIP, FF, PEN, RAC, SAL, SUL, and TAP. The CR values were evaluated with the following equation:

(2) $$\begin{array}{c} CR\left(\;\%\;\right)=\frac{IC_{50}\text{ of }CAP}{{IC}_{50}\text{ of analogue}}\times 100\% \end{array}$$

In this equation, IC_50_ is the half maximal inhibitory concentration of a substance. The specificity results of the anti-CAP mAb were shown in Table 1. From the results, negligible CR values (less than 0.01%) were obtained for all the analogues investigated in this research including the most structure related TAP and FF. It can be concluded that this developed CL-ELISA assay could be applied for specific CAP detection.

### 3.2. Optimization of CL-ELISA Procedure

The antigen to antibody ratio in a competitive reaction system significantly influences the immunoreaction. In this CL-ELISA protocol, the antigen to antibody ratio was evaluated and optimized. Antigen to antibody ratios of 70:30, 60:40, 50:50, 40:60, and 30:70 were investigated. The results were optimized according to IC_50_ and RLUmax (maximum relative light units) and showed in Table 2. The IC_50_ value increased significantly with increasing antibody ratio. When the antigen to antibody ratio was 70:30, the most sensitive result with the lowest IC_50_ value was obtained. Additionally, a high proportion of antibody led to a high RLUmax value. However, for the tested antigen to antibody ratios, all the RLU values were suitable for the detection of CAP. Due to the high sensitivity of chemiluminescent substrate, there was no apparent difference of RLUmax value. In order to obtain the most optimized and sensitive result, an antigen to antibody ratio of 70:30 was adopted in this research.

### 3.3. Sensitivity

**Figure 3:**
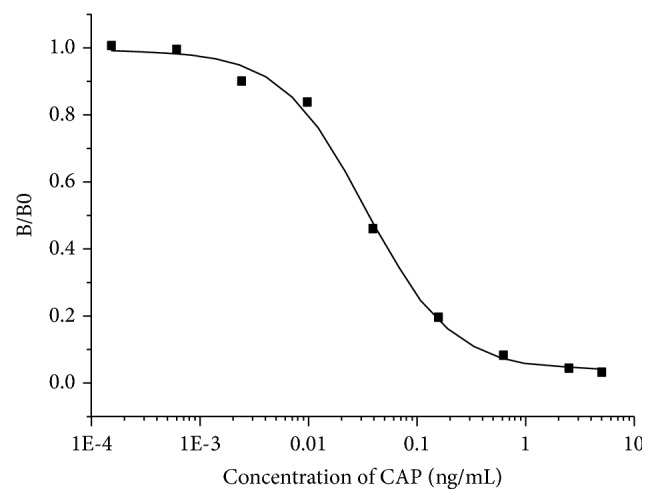
Standard curve for CAP generated from the CL-ELISA under the optimized conditions.

IC_50_, LOD (measured by IC_10_), and the detection range (IC_20_−IC_80_) were obtained from sigmoidal calibration curve to evaluate to sensitivity of the proposed CL-ELISA protocol (Figure 3). In this research, LOD was 0.0021 ng mL^−1^ while the IC_50_ value was 0.016 ng mL^−1^ for CAP. The detection range of this method was 0.00979−0.12026 ng mL^−1^. Compared to established immunoassays, this method exhibited higher sensitivity and lower LOD for CAP [18, 19].

### 3.4. Accuracy and Precision

For accuracy and precision investigation, recoveries of CAP in fortified samples were calculated first. And the accuracy and precision were evaluated with five replicates each on three validation days. At three fortified level of 5, 20, and 100 ng L^−1^, mean recoveries for CAP in spiked primer lotion cosmetics samples ranged from 82.7% to 99.6%. Intraday and interday variation were less than 9.8% and 8.2%, respectively (results were shown in Table 3).

## 4. Conclusions

In this research, a novel and highly sensitive CL-ELISA method for CAP detection in cosmetics was developed. The mAb utilized in the assay exhibited high specificity to CAP with negligible CR values to its analogues, especially for most structure related TAP and FF. On the basis of this very mAb, the proposed CL-ELISA method could be applied for the detection of CAP specifically instead of mixtures of CAP and other analogues. This is the first report of a CL-ELISA for the determination of CAP in cosmetics. The method LOD was 0.0021 ng mL^−1^, IC_50_ was 0.016 ng mL^−1^, and the detection range was 0.00979−0.12026 ng mL^−1^. In spiked cosmetics samples, mean recoveries ranged from 82.7% to 99.6%, with the intraday and interday variation less than 9.8% and 8.2%, respectively. The assay offers the advantages of high specificity, simplicity, fast analysis times, and cost-effectiveness. With respect to its sensitivity and specificity, the developed CL-ELISA method is superior to most reported immunoassays for CAP detection. It can be concluded that this developed CL-ELISA method could be applied for specific, rapid, semiquantitative, and quantitative detection of CAP in cosmetics.

## Figures and Tables

**Table 1:** IC50 and cross-reactivity (CR) value of CAP analogues.

Analogue	IC50 (ng mL^−1^)	CR (100%)
CAP	0.034	100
TAP	>1,000	<0.01
FF	>1,000	<0.01
SAL	>1,000	<0.01
RAC	>1,000	<0.01
SUL	>1,000	<0.01
CIP	>1,000	<0.01
PEN	>1,000	<0.01

**Table 2:** Optimization of antigen antibody ratio.

Antigen antibody ratio	IC 50 (ng mL^−1^)	RLUmax
70/30	0.016	2.11E8
60/40	0.017	2.38E8
50/50	0.021	2.45E8
40/60	0.035	2.68E8
30/70	0.033	2.64E8

**Table 3:** Accuracy and precision of CAP in spiked cosmetic samples.

Analyte	Spiked level (ng L^−1^)	Recovery (%)	Intra-day variation (%)	Inter-day variation (%)
CAP	5	82.7−99.6	9.8	8.2
	20	85.3−98.9	7.9	6.4
	100	88.6−96.9	6.1	5.5

## Data Availability

The data used to support the findings of this study are available from the corresponding author upon request.
